# Methodology for Biological Sample Collection, Processing, and Storage in the Newcastle 1000 Pregnancy Cohort: Protocol for a Longitudinal, Prospective Population-Based Study in Australia

**DOI:** 10.2196/63562

**Published:** 2024-11-15

**Authors:** Joshua J Fisher, Tegan Grace, Nathan A Castles, Elizabeth A Jones, Sarah J Delforce, Alexandra E Peters, Gabrielle K Crombie, Emily C Hoedt, Kirby E Warren, Richard GS Kahl, Jonathan J Hirst, Kirsty G Pringle, Craig E Pennell

**Affiliations:** 1 School of Medicine and Public Health College and Health, Medicine and Wellbeing The University of Newcastle Callaghan Australia; 2 School of Biomedical Sciences and Pharmacy College of Health, Medicine and Wellbeing The University of Newcastle Callaghan Australia; 3 School of Life and Medical Science Faculty of Population Health Sciences University College London London United Kingdom; 4 School of Environmental and Life Sciences College of Engineering, Science and Environment The University of Newcastle Callaghan Australia

**Keywords:** pregnancy cohort study, biobanking protocol, toenails, blood, microbiome, urine, hair, pregnancy, cohort study

## Abstract

**Background:**

Research in the developmental origins of health and disease provides compelling evidence that adverse events during the first 1000 days of life from conception can impact life course health. Despite many decades of research, we still lack a complete understanding of the mechanisms underlying some of these associations. The Newcastle 1000 Study (NEW1000) is a comprehensive, prospective population-based pregnancy cohort study based in Newcastle, New South Wales, Australia, that will recruit pregnant women and their partners at 11-14 weeks’ gestation, with assessments at 20, 28, and 36 weeks; birth; 6 weeks; and 6 months, in order to provide detailed data about the first 1000 days of life to investigate the developmental origins of noncommunicable diseases.

**Objective:**

The study aims to provide a longitudinal multisystem approach to phenotyping, supported by robust clinical data and collection of biological samples in NEW1000.

**Methods:**

This manuscript describes in detail the large variety of samples collected in the study and the method of collection, storage, and utility of the samples in the biobank, with a particular focus on incorporation of the samples into emerging and novel large-scale “-omics” platforms, including the genome, microbiome, epigenome, transcriptome, fragmentome, metabolome, proteome, exposome, and cell-free DNA and RNA. Specifically, this manuscript details the methods used to collect, process, and store biological samples, including maternal, paternal, and fetal blood, microbiome (stool, skin, vaginal, oral), urine, saliva, hair, toenail, placenta, colostrum, and breastmilk.

**Results:**

Recruitment for the study began in March 2021. As of July 2024, 1040 women and 684 partners were enrolled, with 922 infants born. The NEW1000 biobank contains 24,357 plasma aliquots from ethylenediaminetetraacetic acid (EDTA) tubes, 5284 buffy coat aliquots, 4000 plasma aliquots from lithium heparin tubes, 15,884 blood serum aliquots, 2977 PAX RNA tubes, 26,595 urine sample aliquots, 2280 fecal swabs, 17,687 microbiome swabs, 2356 saliva sample aliquots, 1195 breastmilk sample aliquots, 4007 placental tissue aliquots, 2680 hair samples, and 2193 nail samples.

**Conclusions:**

NEW1000 will generate a multigenerational, deeply phenotyped cohort with a comprehensive biobank of samples relevant to a large variety of analyses, including multiple -omics platforms.

**International Registered Report Identifier (IRRID):**

DERR1-10.2196/63562

## Introduction

Research into the developmental origins of health and disease provides compelling evidence that adverse events during the first 1000 days of life from conception can impact life course health, increasing the risk of metabolic risk factors [1,2], such as obesity [3], diabetes [4], hypertension [5], cardiovascular disease [6], asthma [7,8], allergies [9,10], and adverse neurodevelopmental [11,12] and mental health [13-15] outcomes. Despite decades of research, we still lack a complete understanding of the mechanisms underlying some of these associations. Australian pregnancy and birth cohorts have provided excellent contributions to pregnancy health and developmental research over the past 3 decades, with over 17 pregnancy or birth cohorts to date [16]. Since the inception of these studies, there have been significant advances in technologies, increasing our understanding of the underlying biological mechanisms driving the pathogenesis of disease. For example, the rapidly expanding use of “-omics,” such as genomics, transcriptomics, proteomics, and metabolomics, has contributed to identifying complex traits underlying health outcomes with greater accuracy than standard clinical approaches [17].

The Newcastle 1000 Study (NEW1000) is a comprehensive pregnancy cohort study based in Newcastle, New South Wales, Australia, which will provide detailed data about the first 1000 days of life to investigate the developmental origins of noncommunicable diseases [18]. The multiomics design of NEW1000 intends to provide detailed, longitudinal, multisystem phenotyping and serial sample collection for analyses of the genome, microbiome, epigenome, transcriptome, fragmentome, metabolome, proteome, exposome, and cell-free DNA and RNA. A critical element of biorepositories is standardized collection, handling, and storage of samples to ensure reliable results through analyses of high-quality samples [19]. In addition, the use of a suitable informatics system is recommended to ensure accurate tracking of inventory [20]. This manuscript describes the methodology used to collect, process, and store samples in NEW1000, with the intent to facilitate the access and use of these resources upon request.

## Methods

### Study Design

**Figure 1:**
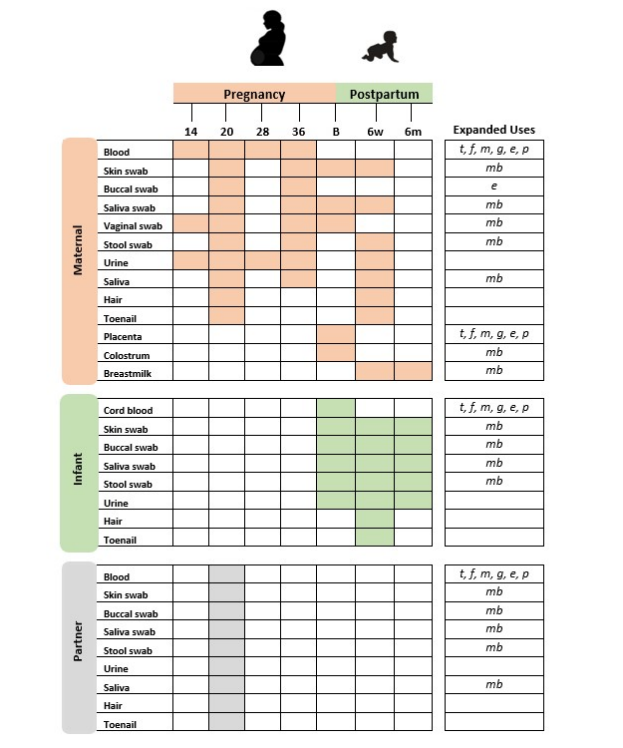
NEW1000 stage 1 biological sample collection timepoints. NEW1000 aims to provide a comprehensive database for use in traditional analysis and emerging -omics platforms. Expanded uses denote the intended platforms and appropriate approaches based on the collection methods highlighted in the manuscript. e: epigenomics; f: fragmentomics; g: genomics; mb: microbiomics; m: metabolomics; NEW1000: Newcastle 1000 Study; p: proteomics; t: transciptomics.

NEW1000 is a prospective, population-based, longitudinal pregnancy cohort study. Stage 1 (2021-2025) will involve recruitment of 500 pregnant participants and their partners per year for 5 years. The importance of the first 1000 days of life from conception is reflected in the number of assessments during this stage. Each family will be seen 5 times during pregnancy (14, 20, 28, 36, and 40 weeks’ gestation), at birth, 1-2 days postnatally, then again 6 weeks, and 6 months postpartum. Stage 2 will involve further postnatal follow-up of the families at 12, 24, and 36 months of age. Stage 3 will involve long-term follow-up of the cohort through face-to-face appointments, questionnaires, and data linkage to establish a deeply phenotyped pregnancy cohort from early gestation through adulthood. The focus of this manuscript will be the protocol and methodology of biological sample collection and biobanking for stage 1 of the study, and recruitment and assessment processes have been previously published [18]. NEW1000 has been designed for standardized collection of blood, urine, saliva, buccal swabs, microbiome swabs, toenails, and hair samples from the mother, father (or partner), and infant. In addition, cord blood, placenta, and colostrum samples will be collected at birth, and breastmilk samples will be collected 6 weeks and 6 months postpartum (Figure 1).

### Blood Samples

The use of human blood within biomedical research, particularly biomarkers, is a significant resource and commonplace in the discovery and diagnosis of clinical conditions. Biomarkers have been defined as “a characteristic that is objectively measured and evaluated as an indicator of normal biological processes, pathogenic processes or pharmacologic responses to a therapeutic intervention” [21]. In addition to the traditional use of biomarkers, there is emerging evidence to support the use of genetic biomarkers for precision medicine—that is, their application for diagnosis, prognosis, and personalized treatment options based on an individual’s genetic profiling [22]. NEW1000 will collect a comprehensive panel of blood and blood products to facilitate all avenues of biomarker research, including traditional approaches and downstream genetic or multiomic analyses. Isolating such biomarkers will allow for the prediction of adult disease in the earlier stages of life and thus provide the potential for advancements in precision medicine.

#### Collection of Blood Samples

Collection of blood samples will be according to the recommended order of draw (Multimedia Appendix 1): the Vacuette serum tube (silica-coated clot activator and polymer gel; Greiner Bio-One) for use in routine clinical chemistry and hormone tests, serology, and immunohematology; the Vacuette heparin plasma tube (lithium heparin coated) used for clinical biochemistry tests; Vacuette ethylenediaminetetraacetic acid (EDTA) tubes (containing K2 EDTA anticoagulant) used for hematological procedures, such as complete blood counts and blood typing and plasma isolation, to allow for multiomics analyses; and the PAXgene blood RNA tube (containing 6.9 mL of RNA stabilization additive; BD Biosciences) used to stabilize intracellular RNA to yield accurate and reproducible gene expression data [23]. Maternal blood samples will be collected at 14-, 20-, 28-, 36-, and 40-week appointments, as well as 6 weeks postpartum. Partner blood samples will be collected at the 20-week appointment, and cord blood will be collected at birth. Adult blood samples to be collected include 8 mL in one 8 mL Vacuette serum tube; 4 mL in one 4 mL Vacuette heparin plasma tube; 18 mL in two 9 mL Vacuette K2 EDTA tubes; and 4 mL of cord blood in one 4 mL Vacuette EDTA tube. The buffy coat, containing white blood cells and platelets, an important source of genomic DNA that can withstand long-term storage [24], will be extracted from centrifuged Vacuette EDTA tubes. In addition to these tubes, 2.5 mL (draw volume) in 1 PAXgene blood RNA tube will be collected for maternal blood samples at 14-, 20-, and 28-weeks’ gestation. Thus, each family will have the potential to donate blood that totals up to 68 aliquots within their first year of the study that can be used for traditional and nontraditional -omics techniques.

#### Adult Blood Samples

Using a tourniquet on the arm above the cubital fossa, a research nurse will locate a suitable vein and clean the site with an alcohol swab. Samples will be collected via a 21G sterile luer and holder with a retractable needle, commonly used during venipuncture as the smaller gauge reduces discomfort and prevents hemolysis. Once all tubes are filled and the needle removed and retracted for safe disposable, a sterile injection pressure pad will be applied to the collection site.

#### Cord Blood Samples

The placenta will be placed in a sterile specimen dish postbirth. If umbilical cord blood gas analysis is required as part of clinical care, it will be obtained prior to research samples. A large cord vein will be located and cleaned using an alcohol swab to remove any maternal blood. An 18G needle will then be inserted into the vein, and a 10 mL syringe will be used to draw a blood sample, which will be put into a 4 mL EDTA tube.

#### Processing and Storage of Blood Samples

**Figure 2:**
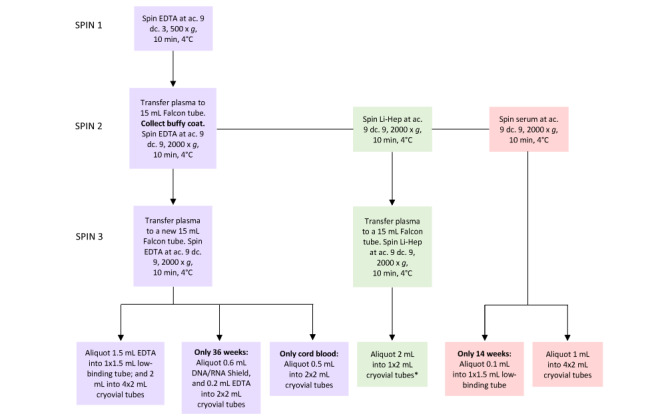
Flow diagram for processing of blood samples. *Lithium heparin plasma can be divided evenly between two 2 mL cryovial tubes if there is >2 mL of plasma. Purple boxes indicate the processing flow of EDTA tubes, green boxes depict lithium heparin tubes, and pink boxes show serum tubes. ac: acceleration; dc: deceleration; EDTA: ethylenediaminetetraacetic acid.

The subsequent sections will discuss the methodology of processing, separation, and storage of plasma, serum, and the buffy coat from the EDTA, serum, lithium heparin, and PAXgene blood tubes (Figure 2).

#### Plasma

**Figure 3:**
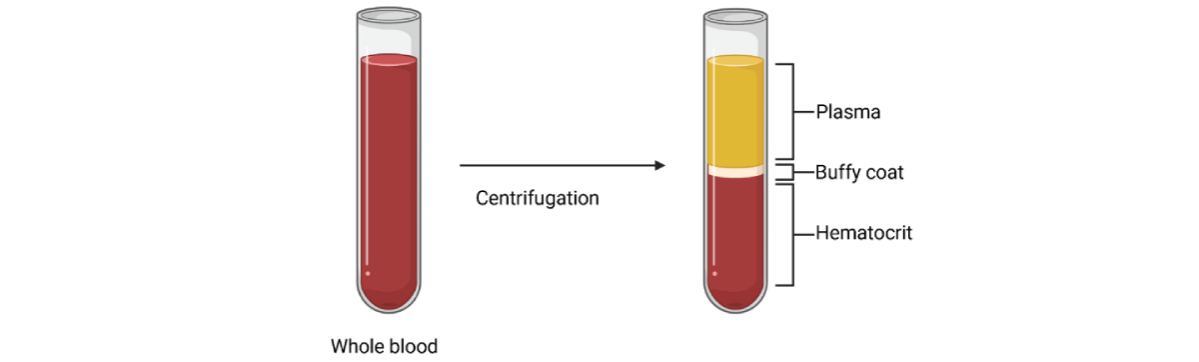
Centrifugation of whole blood to separate blood components (figure created in BioRender): plasma; buffy coat, which contains white blood cells and platelets; and hematocrit (red blood cells).

Samples will be inverted 5-10 times to ensure adequate mixing of blood with the EDTA anticoagulant [25] and centrifuged under the following conditions: 10 minutes at 500 ×*g* at 4 °C, with an acceleration of 9 and a deceleration of 3. All but approximately 0.2 mL of plasma (reserved for the buffy coat; Figure 3) will be aliquoted into one 15 mL Falcon tube and subjected to a second centrifugation step for 10 minutes at 2000 ×*g* at 4 °C, with an acceleration of 9 and a deceleration of 9. Plasma will be aliquoted into a new sterile 15 mL Falcon tube (Corning) and centrifuged again according to the same conditions as the previous spin and the pellet discarded. The remaining plasma will then be aliquoted: 1.5 mL plasma into one 1.5 mL low-binding Eppendorf tube, with the remaining plasma evenly distributed among four 2 mL cryovial tubes. For 36-week appointments, 0.2 mL plasma will be transferred into two 2 mL cryovial tubes containing 0.6 mL of DNA/RNA Shield solution prior to the plasma being distributed into the four 2 mL cryovial tubes. Cord blood samples will be subjected to the same centrifugation conditions; however, a smaller quantity of plasma will be obtained. Approximately 0.5 mL of plasma will be placed into one 1.5 mL low-binding Eppendorf tube and two 2 mL cryovial tubes. When extracting plasma, approximately 0.2 mL will be reserved (in each tube) for the buffy coat. Aliquots will be stored at –80 °C until required for analysis, and the OpenSpecimen database will be updated to include these aliquots.

#### Buffy Coat

A sterile 3 mL Pasteur pipette will be used to aliquot the buffy coat from both 9 mL Vacuette EDTA tubes (one 4 mL Vacuette EDTA tube for cord blood), along with the remaining 0.2 mL of plasma and a small portion of red blood cells into one sterile 2 mL cryovial tube. The tubes will be stored frozen at –80 °C until required for analysis, and OpenSpecimen will be updated to include these aliquots.

#### Serum

Samples will be inverted 5-10 times to activate the silica clotting agent [25] and then kept upright at room temperature for 20 minutes to allow clot formation. Afterward, the samples will be centrifuged under the following conditions: 10 minutes at 2000 ×*g* at 4 °C, with an acceleration of 9 and a deceleration of 9. The serum will be subsequently aliquoted: 1 mL into four 2 mL cryovial tubes and, for 14 weeks’ gestation only, approximately 0.1 mL into one 1.5 mL low-binding Eppendorf tube. Aliquots will be stored frozen at –80 °C until required for analysis, and OpenSpecimen will be updated to include these aliquots.

#### Lithium Heparin

Samples will be inverted 5-10 times to ensure adequate mixing of blood with the heparin anticoagulant [25] and centrifuged under the following conditions: 10 minutes at 2000 ×*g* at 4 °C, with an acceleration of 9 and a deceleration of 9. All the plasma will be aliquoted into one 15 mL Falcon tube and subjected to a second centrifugation spin under the same conditions as the previous spin. All the plasma will then be aliquoted into one 2 mL cryovial tube or divided evenly between two 2 mL cryovial tubes if there is >2 mL of plasma. Aliquots will be stored at –80 °C until required for analysis, and OpenSpecimen will be updated to include these aliquots.

#### PAXgene Blood RNA

Samples will be kept upright at room temperature for a minimum of 2 hours before being stored at –30 °C overnight, and OpenSpecimen will be updated to include the samples. The following morning, the samples will be transferred from –30 °C to permanent storage at –80 °C until required for analysis.

### Urine Samples

Urine can be used for the detection and analysis of proteinuria, bacteria, infections, toxicology, renal function, and nutrient markers [26-28]. Midstream urine samples will be requested at 14, 20, 28, and 36 weeks’ gestation and 6 weeks postpartum from enrolled women. Partner samples will be collected at 20 weeks. Adult samples will be self-collected in 70 mL sterile specimen jars and aliquoted into four 2.0 mL sterile CryoGen CLEARLine cryotubes (Biosigma) for storage at –80 °C. Additional urine will be stored in 15 mL Falcon tubes at –80 °C. Infant urine samples will be collected 6 weeks and 6 months postpartum using a cellulose pad insert to collect urine during appointments. The insert will be collected at the end of the appointment, and urine will be extracted using a specialized press device, then aliquoted into 2.0 mL sterile cryotubes, and stored at –80 °C.

### Saliva Samples

Human saliva is a complex biofluid that is readily available, can be collected noninvasively, and can be used for a number of diagnostic and biomedical research-based analyses [29]. Saliva has been used to study proteomics, with proteins often closely reflecting those seen in plasma, as approximately 30% of salivary proteins originate in the blood [30]. As such, saliva is emerging as a common alternative to blood in genetic research and polymerase chain reaction (PCR)–based genotyping [31]. Further, steroid hormone concentrations and fluctuations are often assessed in salivary samples via enzyme-linked immunosorbent assay (ELISA) or liquid chromatography–mass spectrometry (LC-MS) [32,33]. Salivary cortisol concentrations, routinely studied in animal and human studies, have been validated with serum cortisol concentrations [34-36]. As stressful experiences in early life are linked to numerous adverse developmental outcomes, accurate biospecimen collection for assessment of these pathways within any longitudinal cohort is critical [37,38]. Therefore, saliva will be an important forward-looking and versatile sample within the NEW1000 biobank, providing an often-overlooked biological sample for accessing and validating a multitude of biological markers. Investigating the response to stressors, such as maternal anxiety, depression, and familial and sociodemographic adversity [37,39-41], and external and environmental factors, such as COVID-19, lockdowns, or natural disasters [42,43], will provide a baseline for future long-term programming studies in children and adults.

#### Collection, Processing, and Storage of Saliva Samples

Maternal saliva samples will be self-collected at home on the morning of the 20- and 36-week appointments. Partners will provide a saliva sample at 28 weeks. Participants will be instructed to collect samples within 20 minutes of waking up, before food, drink, or other substances are consumed and before teeth are brushed, to enable assessment of baseline levels of biomarkers and stress hormones, such as cortisol. To maximize the collection amount, participants will be instructed to let saliva accumulate in the mouth without swallowing for 1 minute and then to passively drool the accumulated saliva into a sterile 5 mL screw-cap collection tube (Sarstedt Australia). Samples will be processed by centrifugation in 5 mL collection tubes for 10 minutes at 2000 ×*g* at 4 °C prior to being placed into two 2 mL cryotubes using a sterile 3 mL Pasteur pipette. Duplicate aliquots will be implemented to minimize the need for freeze/thaw and provide an alternative or complimentary examination to blood samples. Samples will be routinely kept at –80 °C for long-term storage [15].

### Buccal Swabs

Recent studies have highlighted the potential advantages offered by buccal swabs when assessing epigenetics, DNA methylation, and genome-wide studies [44-46], most notably the uniformity of the epithelial cell type present compared to other samples [45,46]. Critical to collection of samples for multiomic analyses, buccal samples have recently been used to assess the predictive nature of epigenetics in preterm birth [45], providing a valuable addition to the biobank.

#### Collection, Processing, and Storage of Buccal Swabs

Maternal buccal swabs will be collected at 20 and 36 weeks’ gestation and 6 weeks postpartum; paternal samples at 20 weeks; and infant samples at birth, 6 weeks postpartum, and 6 months postpartum. Prior to collection, adult participants will be asked to refrain from eating, drinking, chewing gum, brushing teeth, or smoking for at least 30 minutes. Participants will be instructed to remove a sterile swab from the tube, ensuring the tip does not touch any surfaces, and rub the swab on both sides of their inner cheek for 60 seconds, rotating the end several times to collect skin cells. Once the collection is completed, the participants will be instructed to remove the swab, being careful the swab tip does not touch the teeth, lips, or any other surface, then place the swab directly into the collection tube, and close the lid firmly. Collection of infant buccal swabs will be completed by a research midwife or nurse.

### Microbiome Samples

The microbiome plays a central role in health and disease, contributing to the development and regulation of metabolic and immune functions [47,48]. From birth, the microbiome changes in response to host and environmental factors [49]. Disruptions in the early life microbiome can negatively impact a wide range of health outcomes [50,51]. Beginning at birth, complex host-microbe-environmental interactions help shape host metabolism, immune and gastrointestinal tract system development, and behavior [47,48], setting the biological foundation for future disease susceptibility [52]. The first colonizers of the infant gut are determined during and immediately after birth when the infant is exposed to microbial communities from the mother and the external environment. The subsequent maturation of the microbiome is dynamic and highly individualized during the first 2 years of life [53]. During this time, multiple factors influence the microbiome structure and function, including the maternal microbiome across surfaces and fluids the infant is exposed to, such as the vagina, skin, saliva, and breastmilk [54].

The maternal microbiome undergoes substantial remodeling during pregnancy that can promote metabolic changes, such as weight gain and insulin insensitivity [55], and promote neonatal growth. Maternal host-microbe interactions contributing to infant health remain poorly understood. Comprehensive studies to address this knowledge gap are critical in order to develop microbiome-targeted strategies to improve pregnancy outcomes and infant health [56]. Although recent studies have provided important insights into microbiome development in early life [51,53,57,58], these studies are limited due to small samples sizes. This cohort aims to alleviate these matters and provide a comprehensive and well-characterized data set using which future studies can elucidate the mechanisms and complex interactions behind maternal and infant microbiomes in fetal development and the onset of disease later in life.

#### Collection, Processing, and Storage of Microbiome Samples

##### Vaginal Microbiome

A self-collected vaginal swab for the vaginal microbiome and a swab of the transvaginal probe cover (postscan) will be requested from each participant, which will provide an upper and a lower vaginal microbiome specimen/sample for assessment. Low vaginal swabs will be self-collected at 14 and 36 weeks’ gestation and during early labor using a standardized health services protocol [55]. Participants will be instructed to wash their hands prior to twisting the cap to break the seal on the sterile swab provided (Copan), being careful the swab end does not touch any other surfaces. With their other hand, they will gently spread the labia and insert the tip of the swab about 5 cm into the vaginal opening, pointing the tip of the swab toward their lower back while relaxing their muscles. The swab should be gently rotated and then held against the vaginal wall for 10-20 seconds. Participants will then remove the swab without it touching the outer skin and return it to its original tube, ensuring the cap is snapped shut. Swabs will be transported immediately to the laboratory and the swab tips cut into 2 mL screw-cap tubes and snap-frozen in liquid nitrogen before being transferred to a –80 °C freezer until extraction [59].

##### Skin Microbiome

Skin microbiome samples will be collected by a sterile swab dipped in 0.15M sodium chloride solution and firmly applied and rotated on the skin for ~60 seconds (chest of the mother/father and cheek of the baby) [60]. Swabs will then be transported immediately to the lab and the swab tips cut into 2 mL screw-cap tubes and snap-frozen in liquid nitrogen before being transferred to a –80 °C freezer until extraction [59]. Maternal skin swabs will be obtained at 20 and 36 weeks’ gestation and at birth; partner swabs at 20 weeks; and infant swabs within 24 hours of birth, 6 weeks postpartum, and 6 months postpartum.

##### Oral Microbiome

Participants will be asked to refrain from eating, drinking, chewing gum, brushing teeth, or smoking for at least 30 minutes before sample collection. Participants will be provided with a sterile swab and asked to collect a saliva sample by rubbing and rotating the swab along the teeth, applying similar pressure as brushing their teeth, and then placing the swab under the tongue for 30 seconds to collect saliva. Participants will be instructed to ensure the swab does not touch their lips or other surfaces prior to placing it back in the sterile tube and securing the lid. Swabs tips will be cut into 2 mL screw-cap tubes and snap-frozen in liquid nitrogen before being transferred to a –80 °C freezer until extraction [59]. Maternal saliva swabs will be obtained at 20 and 36 weeks’ gestation and 6 weeks postpartum, partner swabs at 20 weeks’ gestation, and infant swabs 6 weeks and 6 months postpartum.

##### Gut Microbiome

Collection kits developed by Microba Life Sciences will be used to collect stool samples. This kit consists of an easy-to-use swab and preservation with a liquid-free preservation solution, which is temperature-stable for up to 7 days [61]. A prelabeled swab collection kit will be provided to participants, who will use the sterile swab to collect fecal matter from soiled toilet paper after a bowel movement. Instructions on the amount needed for correct sampling will be provided as part of the self-collection pack. Samples remain stable at room temperature for up to 7 days, allowing participants to collect their sample several days prior to their next appointment and bring the sample in the provided packaging to their next appointment. Maternal fecal swabs will be obtained at 20 and 36 weeks’ gestation; partner swabs at 28 weeks’ gestation; and infant swabs, where possible, within 24 hours of birth, 6 weeks postpartum, and 6 months postpartum. Swabs will be stored in a –80 °C freezer until analysis.

### Measurement of Environmental Exposure

Substances such as endocrine disrupting chemicals, heavy metals, and pesticides are persistent in the environment, leading to human exposure through diet, the household, the workplace, and ambient surroundings [62]. Maternal exposure to environmental contaminants during pregnancy renders the fetus susceptible to indirect exposure and the associated negative health impacts during critical periods of development [63,64]. These health impacts can be diverse and long lasting, with effects including altered human chorionic gonadotropin (hCG) secretion, intrauterine growth restriction, reduced birth weight, and alterations of the neonatal gut microbiome and metabolome [63-66]. Collection and storage of nail and hair clippings for use as a biomarker of environmental contaminant exposure are ideal due to the noninvasive nature of sampling, the ability to assess cumulative exposure, and the ease of long-term storage and handling [67]. Additionally, when substances are deposited into keratin-rich tissues, their levels remain unchanged, allowing for measurement of long-term cumulative exposure over a period of 6-12 months [67,68]. This is of critical importance to the value of these samples as human nails develop at ~10 weeks of gestation. Thus, collection and analysis of infant nail samples early postpartum can enable an estimate of exposure over the span of pregnancy as a whole, compared to blood or urine samples that only provide insight into a short span of time, usually up to a few days [68].

#### Toenails

Analysis of nail samples has proven effective for a range of environmental contaminants, including heavy metals, such as mercury and arsenic; endocrine disrupting chemicals, such as bisphenol A; and other industrial chemicals, such as chlorinated paraffins and per- and polyfluoroalkyl substances (PFASs) [67-71]. Thus far, studies that have used toenail sample analysis in infants have primarily focused on heavy metal exposure, correlating detected levels with maternal dietary exposure from 8 weeks postdelivery through 36 weeks from maternal and infant toenails at prenatal and postpartum time points. Collecting toenail samples to analyze environmental contaminant exposure is a promising yet underused tool, with the detection of an expansive range of harmful contaminants yet to be addressed in the context of fetal exposure and gestational cohorts.

#### Collection, Processing, and Storage of Toenail Samples

Participants will be directed to self-collect toenail clippings after a bath or shower, with cleaned clippers or scissors (maternal and partner samples at 20 weeks’ gestation and maternal and infant samples 6 weeks postpartum). Participants will be instructed to cut toenails as close to the nail bed as comfortable over a clean piece of paper or tissue, with at least 2-3 mm of clippings from each toenail being obtained. The tissue or paper will be folded at the edge of each side to enable ease of transfer into a provided self-sealing envelope to be stored in a dry place at room temperature.

#### Hair Samples

Similar to toenails, hair samples can provide information about toxicology, exposure to heavy metals, harmful chemicals in the environment, and alcohol and illicit substance use. Hair may also be used as a biomarker in chronic stress and certain pathological conditions [72]. As evidenced by the assessment of cortisol concentrations, 3 cm of hair growth from the scalp represents retrospective activity of the hypothalamic-pituitary-adrenal (HPA) axis in the preceding 3 months [73]. A small amount of hair cut close to the scalp is an easily accessible, noninvasive sample that can be obtained with high compliance from many populations [74,75].

#### Collection, Processing, and Storage of Hair Samples

Maternal and partner hair samples will be requested at 20 weeks’ gestation and maternal and infant samples 6 weeks postpartum. Hair will be parted with a comb horizontally on the crown of the head, and a 2-3 mm section of hair below will be sectioned off and, aiming for at least 3 cm of length, cut with sharp hair-cutting shears as close to the scalp as possible. For hair less than 3 cm in length, a 4-5 mm section of hair will be collected. The cut end of the hair will be fastened with medical tape. Samples will be stored, flat, in foil within an envelope and stored in a dry, dark place at room temperature. Hair samples can be stored in this way for several years, with little to no degradation [73-75].

### Placental Samples

**Figure 4:**
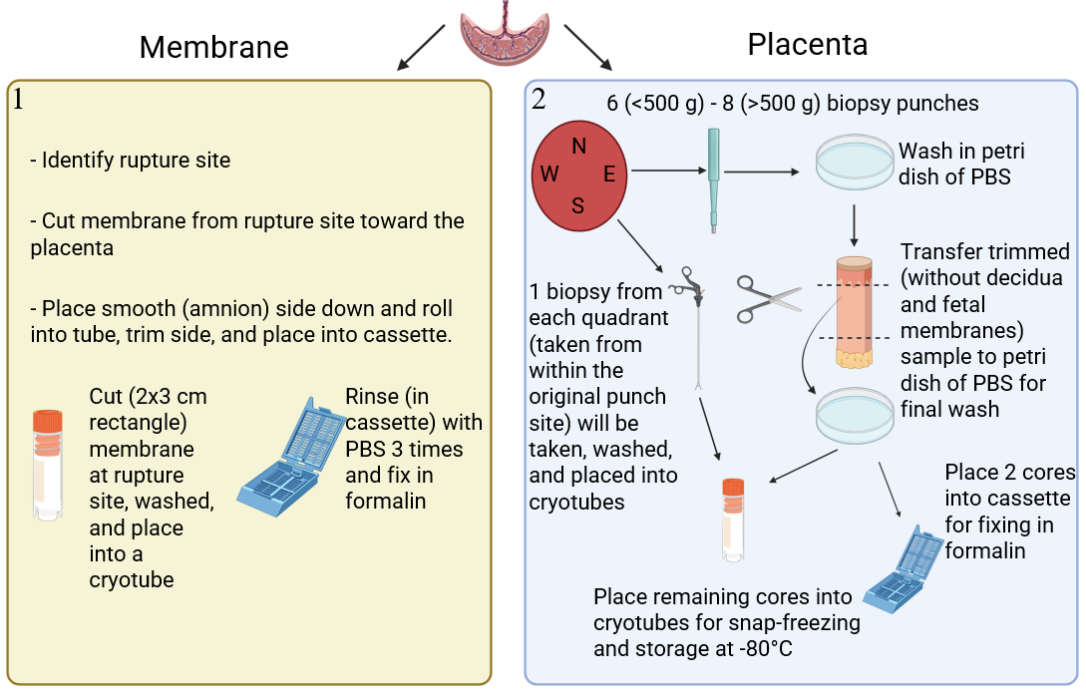
Overview of collection process for placental samples (figure created in Biorender). PBS: phosphate-buffered saline.

Placental samples have previously been used in biomedical research using traditional measures, such as gene and protein work, to interrogate physiology and disease, although rarely in sample sizes cohort studies provide. As methodologies facilitate more comprehensive work, such as genome-wide association studies, proteomics, and metabolomic analysis across more diverse populations, it is critical to maintain high standards of collection and storage to ensure reproducibility. Maintaining accurate and thorough records of placental samples enables the transfer of samples upon request, ensuring use of the cohort, independent evaluation and validation, and creative approaches to reproductive research. As such, in this cohort, we aim to establish a well-characterized and thorough process for consistent and accurate collection to ensure quality. Placentas will be collected as close to birth as possible and taken immediately to the laboratory for processing. Eight individual samples per placenta will be collected, encompassing both the membrane and the villous tissue, in both fixed and frozen format, to obtain the utility and quantity to assess multiple measures per sample (Figure 4).

#### Membrane Collection, Processing, and Storage of Placental Samples

Placental weight and processing time will be recorded. As the membrane must be repositioned or removed prior to placental villous core sampling, the fetal membrane will be collected first. The site of rupture will be ascertained and a cut made posteriorly to collect a 2 × 3 cm^2^ section, which will immediately be washed in ice-cold phosphate-buffered saline (PBS) tablets (Gibco) at pH 7.45) and then dabbed on Kimwipes (Kimtech) to remove excess PBS prior to being snap-frozen in 2 mL cryotubes. For subsequent storage, samples will be frozen at –80 °C until required for analysis. In addition to frozen samples, a section of rolled membrane approximately 2 × 5 cm^2^ will be obtained from the rupture site. This membrane strip will be washed in ice-cold PBS prior to placing the amnion face down on a Kimwipe, and forceps will be used to gently roll the sample. Each edge of the membrane roll will then be subjected to trimming with scissors and placed into an immunohistology embedding cassette (TechnoPlas). The cassette containing the fetal membrane roll will then be placed into a 70 mL specimen pot and subsequently washed 3 times in PBS and placed on a rocker in formalin at 4 °C overnight. Following fixation, formalin will be removed and replaced with a PBS/sodium azide solution (0.1M PBS with 0.05% sodium azide) to prevent bacterial growth for long-term storage. Fixed samples will be stored for the long term at 4 °C.

#### Villous Collection, Processing, and Storage of Placental Samples

The number of samples obtained from the placenta will be determined by the weight of the placenta and whether clinical histopathology examination has been requested. Placentas<500 g will have a maximum of 6 sample cores, while in placentas>500 g, up to 8 cores may be collected*.* This is to ensure that all placental integrity can be maintained upon returning the placenta to histopathology for subsequent clinical macro- and microscopic evaluation. Core samples will be obtained numerically from each of the 4 quadrants (north, south, east, and west) of the placenta (Figure 4) in a process adapted from Burton et al [76]. Cores will be gathered by an 8 mm biopsy punch (Kai) and immediately placed in a petri dish filled with ice-cold PBS, ensuring that the samples are washed gently and thoroughly to minimize maternal blood carryover. Samples will be transferred to a new petri dish containing ice-cold PBS, where scissors will be used to exclude the fetal and maternal membranes. Following this, 4-6 samples (depending on placental weight) will be placed into 1 screw-cap 2 mL cryotube and snap-frozen in liquid nitrogen. Due to limited samples, tissue will not be homogenized immediately prior to being frozen, although we will form a representative cross section once the samples are frozen within the tubes. Tubes will be stored frozen at –80 °C until required for analysis. The remaining core samples will be placed in immunohistology embedding cassettes and fixed and stored, as described before. In addition to whole placental samples, individual biopsies will be obtained from the initial core sites via Klini Tischler (3 × 7 mm^2^ jaw) biopsy tool. Samples will be rinsed in PBS, dabbed dry, and placed in uniquely labeled and barcoded individual Eppendorf tubes (1.5 mL) to represent their respective quarter.

### Biobanking

NEW1000 is a large-scale cohort study. Each family will donate dozens of biological samples, thus contributing to an extensive biospecimen data set. One family could donate up to 150 aliquots of biospecimens within their first year, equating to 75,000 aliquots for the first 500 families. The need to accurately and efficiently store and retrieve samples for subsequent analysis is critical. The NEW1000 biobank will ensure accurate processing, storage, and tracking of all specimens. “Biobanking” is a term used to describe large-scale biospecimen collection and storage within a biorepository [77]. For effective management, biobanking software—known as the laboratory information management system—can be implemented. One such platform that uses this software is OpenSpecimen, an open source database that aims to ensure that extensive sample collection, research, and derivation of meaningful data are as streamlined as possible, allowing researchers to obtain high-quality biospecimen data [78]. As OpenSpecimen is highly configurable, it provides a customizable platform that will suit the needs of the study. Each participant will have a deidentified participant identification number and a profile that contains all relevant medical information pertaining to each gestational time point. All biospecimens collected as part of the study will be barcoded and scanned into the NEW1000 biobank according to each participant’s visit date. Additionally, OpenSpecimen can be integrated with other applications or instruments, such as REDCap [78]. This will ensure that all biospecimens are accounted for and easily retrieved upon request for future studies.

### Ethical Considerations

This study was approved by the Hunter New England Local Health District Human Research Ethics Committee (December 8, 2020; 2020/ETH/02881). All participants provided written informed consent. All proposed projects using NEW1000 data or biological samples will be required to submit an initial application to the NEW1000 Executive Committee and have relevant ethics and safety clearances from the Hunter New England Local Health District Human Research Ethics Committee prior to the release of any deidentified data or biological samples.

## Results

Recruitment for the study began in March 2021. As of July 2024, 1040 women and 684 partners were enrolled, with 922 infants born. The NEW1000 biobank contains 24,357 plasma aliquots from EDTA tubes, 5284 buffy coat aliquots, 4000 plasma aliquots from lithium heparin tubes, 15,884 blood serum aliquots, 2977 PAX RNA tubes, 26,595 urine sample aliquots, 2280 fecal swabs, 17,687 microbiome swabs, 2356 saliva sample aliquots, 1195 breastmilk sample aliquots, 4007 placental tissue aliquots, 2680 hair samples, and 2193 nail samples.

## Discussion

### Summary

NEW1000 provides a robust and deeply characterized cohort with a comprehensive biobank of samples relevant to a large variety of -omics platforms. This manuscript provides a detailed description of collection and storage procedures used within the NEW1000 cohort. Study design, consumer engagement, demographics of the participants, study setting, governance structure, recruitment process, inclusion and exclusion criteria, and nonbiological data collection in the form of questionaries are discussed elsewhere [18]. The breadth and depth of biological samples and data have the potential to generate new and exciting research outcomes and improve the health of future generations. Potential uses for the data include investigations into the early origins of disease, with data collected from early pregnancy through 6 months within the current stage of the study. Planned follow-up of the cohort at 12, 24, and 36 months is underway, with a focus on child development.

### Conclusion

The Newcastle 1000 Study (New1000) is designed as a resource for collaboration, with data linkage, or implementation of a substudy within the cohort, including a new clinical trial, observational study, or intervention. Applications to access New1000 are highly encouraged and outlined in depth [79]. All proposed research projects will be subject to a review process prior to approval. Each application will be reviewed by the NEW1000 Management Group and Executive Committee. The NEW1000 Executive Committee meets face to face on a quarterly basis; however, if there is a need for a project submission to be reviewed outside of this time, frame reviews can be completed electronically. Please allow 6-8 weeks for an application to be reviewed. Data and biosample access requests will be reviewed fortnightly by the study management group. All data and biosample access requests will be cross-referenced with project forms and ethics approvals. If approved, the study manager will liaise with the study data manager and the lead investigator to arrange transfer of deidentified data and samples. The time of data extraction and transfer may vary according to the number and type of data and samples requested.

## Appendix

Multimedia Appendix 1 "Order of draw" chart.

## Abbreviations

EDTA: ethylenediaminetetraacetic acid
ELISA: enzyme-linked immunosorbent assay
LC-MS: liquid chromatography–mass spectrometry
NEW1000: Newcastle 1000 Study
PBS: phosphate-buffered saline
