# Supplementary material for: Methodology for Biological Sample Collection, Processing, and Storage in the Newcastle 1000 Pregnancy Cohort: Protocol for a Longitudinal, Prospective Population-Based Study in Australia
Source: JMIR Res Protoc. 2024 Nov 15;13:e63562. doi: 10.2196/63562 (PMC11607578; doi:10.2196/63562)
Supplement: Multimedia Appendix 1 [file resprot_v13i1e63562_app1.pdf]

NSW Health Pathology

NSWHP_SD_086

ORDER OF DRAW - BLOOD COLLECTION

Recommended order of draw based on CLSI GP41-ED7

Paediatrics: Be aware of sample volume in relation to patient weight and collection frequency

Sodium Citrate: Discard tube required when collecting with a wingset

NSW GOVERNMENT

NOTE

If unsure use the NSW Health Pathology Test Catalogue

TEST CATALOGUE

The volume of blood taken should be age appropriate and minimal. Please consider frequency of collections.

ORDER OF DRAW	TUBE CONTENTS	TEST GUIDE<br>Tests below are a guide, confirm local requirements in NSW Health Pathology Test Catalogue	
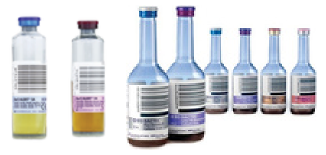	Blood Culture Bottles Aerobic (blue) Anaerobic (purple)	Routine Blood Culture (paired bottles for adults and single for Pediatric) Fungal and Myco Lytic (single bottle)	
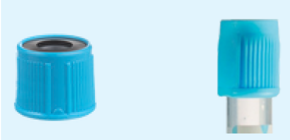	Sodium Citrate	Coagulation Studies, INR, APTT, PT, Fibrinogen, D-Dimer<br>Special tests: Protein C & S, AT3, Lupus, Factor studies<br>Tube must be filled to within the arrow 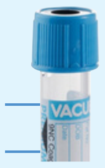	INVERT 4-5 TIMES
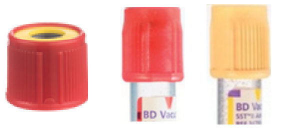	Serum<br>*Serum + Gel	Chemistry, Serology, Immunology, Endocrinology, Rheumatology Routine, non-urgent Biochemistry, Drug levels	INVERT 5-10 TIMES
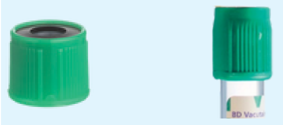	Li Heparin<br>*Li Heparin + Gel	Karyotype, Amino Acids**, Metanephrine** and Levetiracetam Level<br>All urgent biochemsitry: ED, ICU CCU, local to test lab<br>Troponin where assay allows, Cytogenetics	INVERT 5-10 TIMES
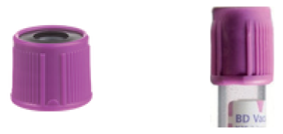	EDTA	FBC, HbA1C, Retics, Ciclosporin Level, Kleihauer, G6PD,<br>Dedicated tube for :<br>ALL PCR tests (1 tube collected is enough),<br>Flow Cytometry, Ammonia **	INVERT 5-10 TIMES
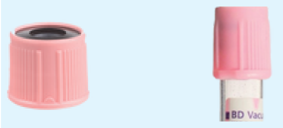	EDTA - Transfusion	Blood group, Group & Hold, Crossmatch, Group Antibody Screen Neonatal, Direct Coombs, Antenatal screening	INVERT 5-10 TIMES
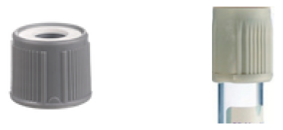	Fluoride Oxalate	GTT, Glucose (stimulation tests)<br>Lactate**, Beta Hydroxybutyrate**, Free fatty acid**	INVERT 5-10 TIMES
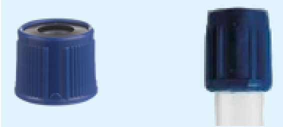	Sodium Heparin	Trace Metals and Heavy Metals	INVERT 5-10 TIMES
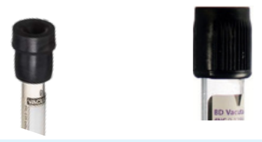	Sodium Citrate	ESR	INVERT 5-10 TIMES
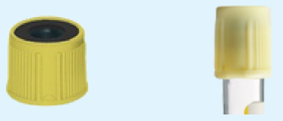	ACD	Tissue typing (special)	INVERT 5-10 TIMES
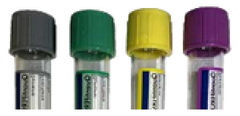	Quantiferon Gold Kit	Tuberculosis	INVERT 5-10 TIMES

IMPORTANT

THOROUGH MIXING
Is necessary to ensure adequate performance of the additive with the blood sample.

DO NOT SHAKE
Gently invert all tubes 5-10 times. Cooagulation tubes 4-5).

FILL MARK
Hold tube in place until filled to the required level

IDENTIFICATION
Make sure to identify the patient and sample correctly.

*Some tests must be collected into a tube without gel. Refer to NSW Health Pathology Test Catalogue
** These tests may be required to be placed on Ice slurry and delivered to Laboratory. Confirm in NSW Health Pathology Test Catalogue

Approver: Director Operations, Version Number: V1, Publication Date: 20/05/2024
